# Development of a lead foil crown delineation technique for implant rehabilitations to generate patient specific finite element model of occlusal loading points

**DOI:** 10.1016/j.mex.2021.101373

**Published:** 2021-05-03

**Authors:** Shyam Sundar S, Sahith Kumar Shetty, Ganesh S

**Affiliations:** a Department of Oral and Maxillofacial Surgery, JSS Dental College and Hospital, JSS Academy of Higher Education and Research, SS Nagar, Mysore 570015, India; b Department of Prosthodontics, JSS Dental College and Hospital, JSS Academy of Higher Education and Research, SS Nagar, Mysore 570015, India

**Keywords:** Functional rehabilitation, Patient specific finite element model, Occlusal surface model

## Abstract

Understanding the clinical biomechanical basis of dental implant supported functional rehabilitation of edentulous jaws improves precision, longevity and overall success of a planned treatment. Stress distribution pattern around dental implants is an important determinant for rate of bone resorption around them. During planning the treatment for most prosthetic rehabilitations, the surgeon uses a software to virtually plan the dimension, position and angulation of the implants considering only the quantity of available bone in the area of interest but does not usually consider the strain generated around the implants after prosthetically loading them. We hence hypothesise that dental implants not be subjected to abnormal strain they should be positioned and angulated not only based on volume of bone available but also based on the vector of occlusal load. The virtual FEA model to analyse the stress distribution would hence require alveolar bone with future tooth/ teeth in centric relation to be modelled. This paper proposes a simple innovative technique to develop a 3D FE model of occlusal loading surface by using a radio-opaque malleable lead foil to generate a patient specific FE model. This would greatly minimise modelling errors and also help determine the best position of the dental implant based on both the volume of bone in the CT scan and the results of FE analyses.

• *Functional rehabilitation using dental implant supported prosthesis needs to be biomechanically analysed to know and understand the stress distribution pattern around the implant.*
• *When teeth (Loading points) are missing, patient specific virtual model of occlusal loading points cannot be generated.*
• *‘Lead foil crown delineation technique’ helps to generate patient specific 3D model of occlusal surface for load application*.

## Graphical abstract

**Figure:**
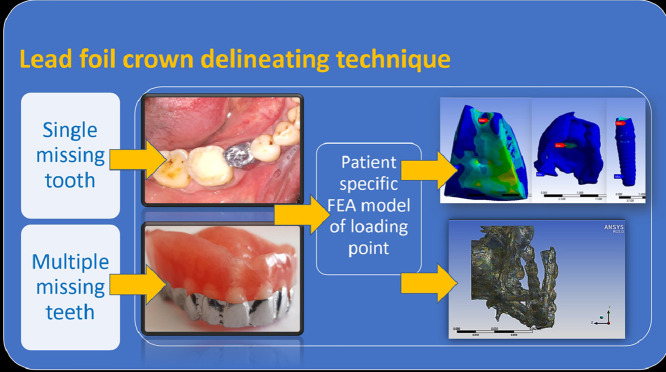

Specifications table

**Table:** 

Subject Area:	Medicine and Dentistry
More specific subject area:	*Oral and Maxillofacial Surgery*
Method name:	*Lead foil crown delineation technique*
Name and reference of original method:	*Not applicable*
Resource availability:	*Not applicable*

*Method details

## Understanding the existing problem and the solution in ‘lead foil crown delineation technique’

The dental implants are often positioned in the jaws using 3D printed implant positioning surgical guides. The surgeon uses the software to plan the angulation and position of the implants considering only the quantity of available bone in the area of interest but not the strain which would be generated around the positioned dental implants, a vital factor for long-term success of the treatment executed.

**Fig. 1:**
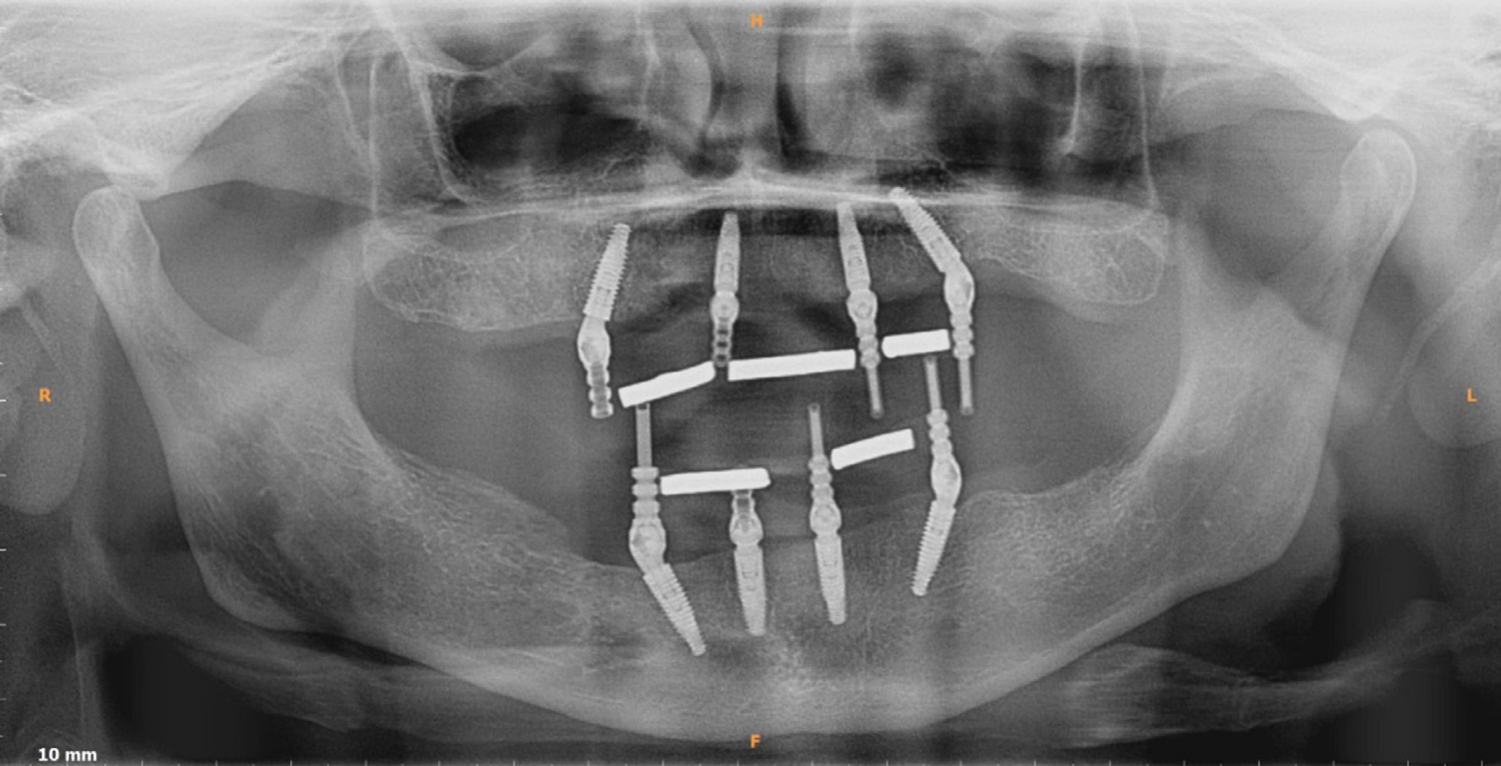
Position and angulation of dental implants in ‘All on 4’® concept in upper and lower jaw.

During the replacement of a single missing tooth, loading vectors are usually along the long axis of the implant [1]. However, the case is different in treatment planning for a full mouth rehabilitation in patients who are completely edentulous, where the ‘All on 4’® concept with tilted distal implants are used widely among implantologists. The basic design of the ‘All on 4’® concept consists of 2 anterior axial and 2 distal angulated implants, over which a bar attached artificial denture is fabricated [2,3] (Fig. 1). The loading vectors on the distal angulated implants are at an angle away from the long axis of the implant and hence ignoring the strain generated around the implant can become problematic when determining the position of an angulated implant based only on the volume of bone [4].

Hence, we hypothesise that dental implants not be subjected to abnormal strain, it should be positioned and angulated not only based on volume of bone available but also based on the vector of occlusal load.

The problem though is to relate the loading vector to the strain generated around the implants. The only logical way is to relate the position of teeth (loading point) and the available bone for implant placement. When there is a single tooth or multiple teeth missing, it is not possible to model load application point. It requires a patient specific FEA analyses of a virtual model of the alveolar bone with future tooth/ teeth in centric relation, which is a customised position of teeth in the patient's upper and lower jaw at rest.

This paper proposes a simple innovative technique to develop a 3D FE model by delineating the occlusal loading points using a radio-opaque malleable lead foil and then generate a patient specific FE model to determine the best position of the dental implant based on both the volume of bone in the CT scan and the results of FE analyses.

## Methodology

**Fig. 2:**
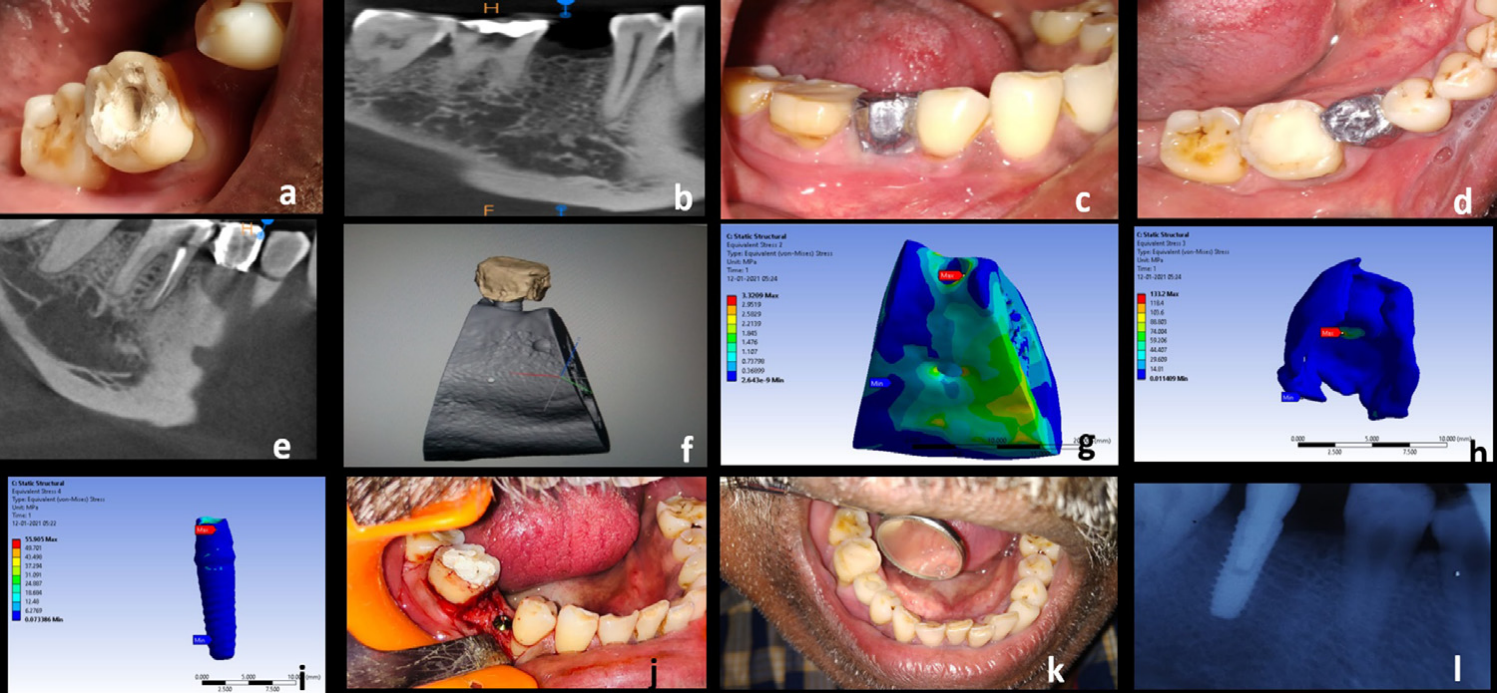
Clinical application process of implant supported single tooth rehabilitation in a respective patient, using lead foil crown delineation technique. a) Single missing tooth. b) CBCT of single tooth missing region. c) Removable prosthesis with countered led foil – buccal view. d) Removable prosthesis with countered led foil – occlusal view. e) BCT delineating the radiopaque lead foil crown. f) 3D model generated. g) FE analysd model of alveolar bone. h) FE analysed model of tooth crown. i) FE analysed model of dental implant. j) Implant placement. k) Final prosthesis on implant. l) Post operative radiograph.

**Fig. 3:**
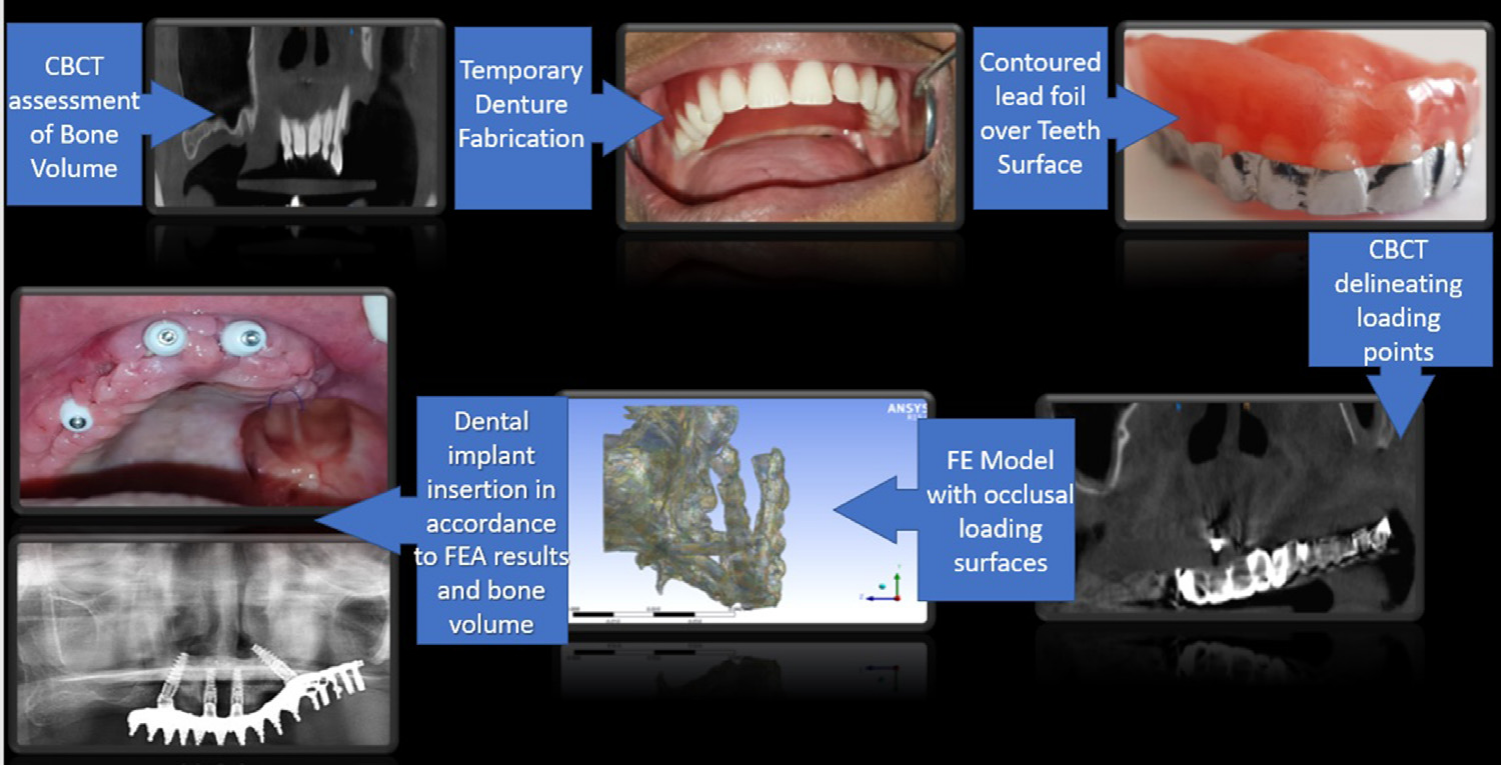
Clinically applied process of implant supported rehabilitation of completely edentulous jaws in the respective patient using lead foil crown delineation technique.

The proposed process is executed after an informed consent from the patients requiring functional rehabilitation. Practical clinical prosthetic problems for functional rehabilitations of jaws are of two kinds. One rehabilitating a single missing tooth and other multiple missing teeth/completely edentulous patient. This novel method of generating FE model using this lead foil is a versatile technique and is applicable to both scenarios. The current proposed procedure has been clinically applied in 4 patients requiring single tooth replacements in the four quadrants of the mouth (maxillary anterior, maxillary posterior, mandibular anterior and mandibular posterior regions) and one patient requiring full maxillary arch rehabilitation. The material properties assigned during creation of FE model is given in Table 1. The process is explained theoretically in Table 2 and the clinical application of the same has been demonstrated by series of photographs involving the preoperative, method of model generation and postoperative phases (Figs. 2a–l, 3).

**Table 1:** Material properties assigned to different material compounds during creation of FE model [5,6].

Material	Elastic modulus (E) (Gpa)	Poisson's ratio (µ)
Titanium (implant)	110^27^	0.35
Cancellous bone	1.37^28^	0.3
Cortical bone	13.7^28^	0.3
Feldspathic porcelain	82.8^30^	0.35
Polymethyl methacrylate	2.4	0.2

**Table 2:** Methodology description.

S. no	Description of method	Need	Single tooth missing	Completely edentulous Jaw
Step 1	CBCT – (Cone Beam Computerised Tomography) DICOM (Digital Imaging in Communications and Medicine) format files generated with resolution of 0.29 mm × 0.29 mm and a slice thickness of 0.6 mm	Bone Volume assessment.	DICOM image of alveolar bone and basal bone in the area of missing tooth which needs to be rehabilitated obtained	DICOM image of alveolar bone and basal bone of the entire edentulous jaw which needs to be rehabilitated obtained
Step 2	Polymethyl methacrylate (PMMA) prosthetic tooth/teeth fabrication	Determination of patient specific anatomical position of future tooth/ teeth.	Fabricate and position the prosthetic tooth in occlusion with opposing jaw teeth under strict aseptic precautions.	Fabricate a complete denture with teeth in centric relation (customised position of teeth in the patient's upper and lower jaw at rest).
Step 3	Contouring of lead foil over crown of prosthetic teeth. Materials Required: Malleable lead foil of 0.5 mm thickness, Wax carver, Surgical knife No.11, B.P handle No.3, Glue (Non cyanoacrylate)	Radio opaque delineation of tooth surface in CBCT. PMMA is otherwise radiolucent.	Disinfect and contour the foil outside the patient's mouth. Adapt and glue the foil without any overlap and not extending on to gums or adjacent teeth. Position correctly in occlusion into the clinically missing tooth space under strict aseptic precautions	Contouring procedure similar to what is done over single teeth. Extend the adaptation to all teeth fabricated, not extending over the denture flanges. Insert the lead foil contoured denture onto the edentulous jaws.
Step 4	CBCT – DICOM (format files generated with resolution of 0.29 mm × 0.29 mm and a slice thickness of 0.6 mm.	CBCT of edentulous area/jaw along with the countered lead foil on the crown is made so that DICOM image of the delineated radio opaque patient specific occlusal surface obtained.	Delineated radio opaque single tooth crown surface obtained. Other teeth appear less radio opaque than lead foil.	Delineated radio opaque crown surface of all teeth in complete denture obtained. Denture flanges are radiolucent
Step 5	Generation of 3D assembly: DICOM images imported visualised, segmented and rendered using segmentation software (MIMICS - Materialise's Interactive Medical Image Control System).	Presurgical model generation (Alveolar bone with implant and crown) to be done as per the objectives of the study (central fossa/buccal /lingual cusp) such that the occlusal load is transferred to the implant and the alveolar bone during (FE analyses which is to be done next) to study the stress distribution pattern in the implant and the alveolar bone surrounding it	Virtual implant of customised dimension is modelled (in connection with the occlusal loading surface) into the best possible position and angulation determined by the volume of bone available	Virtual implants of customised dimensions are inserted into the best possible position and angulation determined by the volume of bone available. A 2mm cylindrical bar connecting all implants and the virtual occlusal surface is modelled (assigning the material properties of titanium), replicating the laboratory method of fabrication.
Step 6	Finite element analyses: Segmented and meshed anisotropic 3D solid model (assigned material properties in accordance to Table 1) is biomechanically analysed using FE software (Abaqus/CAE® (v6.13-1, Dassault Systèmes Simulia Corp., Providence, Rhode Island, USA)	Since the model of the crown is anatomically precise the points of load application could be varied as per the objectives of the study (central fossa/buccal /lingual cusp). Von Mises stress distribution pattern in and around the implant is analysed for loads of varying magnitude and directions. The best dimension, angulation and position of dental implant is then determined.	Tooth Crown modelled using lead foil crown delineation technique is assigned material properties of feldspathic Porcelain / PMMA to mimic a crown customised for patient specific needs.	Tooth crown modelled using lead foil crown delineation technique is assigned the property of PMMA/feldspathic porcelain to mimic patient specific needs.
Step 7	Surgical implant placement using 3D printed surgical guides, into alveolar bone.	Implants positioned and angulated into the bone based on both the volume of bone available and results of FE analyses would increase the stability, longevity and overall success of planned treatment.	Biomechanically analysed single implant supported prosthetic crown. The position to be checked radiographically during the postoperative period	Biomechanically analysed dental implant supported prosthetic rehabilitation of completely edentulous jaws, based on ‘All on 4’® treatment concept. The position to be checked radiographically during the postoperative period

## Advantages of the lead foil crown delineation technique

The entire concept behind the generation of this technique is to reduce the modelling errors as much as possible. It mimics the reality by incorporating the real material properties and as much architectural features as possible so that results achieved after FE analyses would mimic real-time biomechanical scenario and hence highly reliable. The masticatory apparatus of every human is unique and hence it generates anatomically specific loads of varying magnitude and direction. Every point load on the facial skeleton be it on the maxilla, mandible or surface of teeth, generate unique stress pattern, based on which rehabilitation is to be planned.

The precision with which the FE models are generated by this method is very much appreciable. The occlusal surface models depict accurate anatomy mimicking reality. Hence it gives an opportunity for the researcher to customise load application points to individual teeth cuspal levels so that loading conditions can also be modified by altering the design of cusps of individual teeth in case of increased strain noted around the dental implants on loading on specific occlusal points. The technique is inexpensive, does not require high skill or training. It is reproducible under various scenarios with similar clinical problems, requiring biomechanical 3D FE model generation for customised load application points.

A possible disadvantage of this current proposed procedure is the requirement of 2 CBCT examinations during the complete treatment. An improvisation of the current proposed protocol would be to take a CBCT using the existing removable prosthesis for generation the FEA model of the occlusal loading points using the lead foil crown delineation technique along with assessment of bone volume and custom fabricate surgical stent to position the implants in accordance to the FEA results. This would hence reduce the requirement from 2 CBCT to a single CBCT as proposed in the current protocol.

## Conclusion

The success of any functional rehabilitation is measured in terms of its precision, longevity and efficacy. Surgeon planning for rehabilitation, should be aware of the biomechanical perspectives of treatment planning. The biomechanical engineer should understand the bone biomechanics from macro and micro perspectives so that rehabilitation withstands multidimensional load. The proposed patient specific FE model generation of occlusal load application points using lead foil crown delineation technique is a precise, inexpensive, versatile and simple solution in scenarios requiring complex virtual simulations of varied loading points to mimic reality. Thus, the accuracy of finite element analyses would increase with customised patient specific model generation.

Even though the proposed technique aims to optimise loading around dental implants based on FE analyses to minimise bone loss during function, a study is required to compare the results of the proposed procedure to conventional implant based on volume of bone alone.

**Additional material:**

**Biomechanical considerations in functional treatment planning using dental implants**

For a patient requiring maxillofacial functional rehabilitation with dental implants, its architectural complexity should be seen from both the surgeon's and bioengineer's perspective. Such multidisciplinary approach would provide a customised comprehensive rehabilitative solution. Precision, efficacy and longevity of functional rehabilitation of jaws are dependent on both primary mechanical stability of a dental implant and osseointergration. The position and angulation of a dental implant determines the stress distribution pattern for a given load around it which over a period of time determines the pattern of bone resorption around it and hence the stability of an implant [7].

## Declaration of competing interest

The authors declare that they have no known competing financial interests or personal relationships that could have appeared to influence the work reported in this paper.
